# Shotgun metagenomic sequencing for detection of foodborne pathogens in retail chicken

**DOI:** 10.1128/mra.00033-25

**Published:** 2025-05-22

**Authors:** Anuradha J. Punchihewage-Don, Nur A. Hasan, Salina Parveen

**Affiliations:** 1 Food Microbiology and Safety Laboratory, Department of Agriculture, Food and Resource Sciences, University of Maryland Eastern Shore 14705, Princess Anne, Maryland, USA; 2 EzBiome Inc., Gaithersburg, Maryland, USA; Montana State University, Bozeman, Montana, USA

**Keywords:** shotgun, metagenomic, sequencing, foodborne, pathogens, retail chicken, pre-enrichment, whole carcass enrichment, whole carcass rinse, *Salmonella*

## Abstract

We applied shotgun metagenomic sequencing to microbiomes from retail chicken processed using whole carcass enrichment and rinse methodologies to evaluate their effectiveness in detecting foodborne pathogens. The dataset has been made publicly available to facilitate future analysis of microbial diversity and pathogen presence across different sample processing methods.

## ANNOUNCEMENT

Chicken is a well-documented source of foodborne pathogens, including *Salmonella* (1–5). To avoid *Salmonella* outbreaks, the United States Department of Agriculture - Food Safety and Inspection Service (USDA-FSIS) recommends testing for *Salmonella* using whole carcass enrichment (WCE) and rinse (WCR) methods (6–8). This study evaluates the efficiency of WCE and WCR methods in detecting pathogens in retail chicken using shotgun metagenomic sequencing.

Whole broiler carcasses (*n* = 5) were purchased from a retail store in Maryland (38.420904° N, 75.563683° W) and processed as described previously (9, 10). Each carcass was shaken with 500 mL of Buffered Peptone Water (BPW) and separated as follows: 30 mL of rinsate mixed with 30 mL of sterilized BPW for WCR, 100 mL reserved for pre-enrichment, and the remaining liquid along with the carcass designated for the WCE. Samples for both WCE and WCR were incubated at 37°C for 24 hours. Fifty milliliters of incubated WCE, WCR, and 100 mL of pre-enrichment were filtered through 1 micron and 0.22 micron filters using a mechanical pump and Sterivex unit. DNA was extracted from the filters using the DNeasy PowerWater Kit (Qiagen, USA) according to the manufacturer’s protocol.

DNA libraries (1 ng/sample) were prepared using the Nextera XT DNA Library Preparation Kit (Illumina, USA) following the manufacturer’s protocol. Samples were indexed with IDT Unique Dual Indexes (IDT, USA) and amplified via 12 PCR cycles followed by purification with AMPure Beads (Beckman Coulter). Purified libraries were then eluted in Qiagen EB buffer, quantified with Qubit dsDNA HS Kit (Thermo Fisher Scientific), and sequenced on an Illumina HiSeq X platform (2 × 150 bp), targeting 20–40 million read depth per sample. The raw sequencing reads were quality checked using Galaxy’s FastQC (version 0.12.1) with default settings (11). Sequencing data had an average guanine and cytosine (GC) content of 52.78% (range: 43.96%–59.73%), with reads ranging from 14.6 to 62.2 million (average: 21 million), and bases ranged from 4.4 to 18.8 billion (average: 6.3 billion; Table 1).

**TABLE 1:** Sequence Read Archive (SRA) accession and BioSample information for shotgun sequencing data of retail chickens

SRA accession	Biosample accession	Sample ID	Total number of reads	Total number of bases	GC (%)
SRR31730426	SAMN45806799	215A	18,785,235	5,673,140,970	46.45
SRR31730427	SAMN45806798	210R	24,457,973	7,386,307,846	58.31
SRR31730428	SAMN45806797	210E	19,570,162	5,910,188,924	55.96
SRR31730429	SAMN45806796	210A	16,189,223	4,889,145,346	43.96
SRR31730430	SAMN45806795	207R	20,019,104	6,045,769,408	59.73
SRR31730431	SAMN45806794	207E	16,502,962	4,983,894,524	54.5
SRR31730432	SAMN45806793	207A	14,826,135	4,477,492,770	49.72
SRR31730433	SAMN45806792	202R	14,616,232	4,414,102,064	54.44
SRR31730434	SAMN45806804	219R	15,633,744	4,721,390,688	58.65
SRR31730435	SAMN45806803	219E	16,946,670	5,117,894,340	55.1
SRR31730436	SAMN45806802	219A	18,951,763	5,723,434,226	48.07
SRR31730437	SAMN45806801	215R	20,988,239	6,338,448,178	55.25
SRR31730438	SAMN45806800	215E	14,910,873	4,503,083,646	54.35
SRR31730439	SAMN45806791	202E	19,988,909	6,036,450,898	52.26
SRR31730440	SAMN45806790	202A	62,231,035	18,793,772,570	44.97

## Data Availability

The shotgun sequencing data generated for this study have been deposited in the NCBI Sequence Read Archive (SRA) and publicly available through BioProject accession number PRJNA1197471 (https://www.ncbi.nlm.nih.gov/bioproject/PRJNA1197471). The individual SRA accession numbers are included in Table 1.
